# Recent advances in hydrogen peroxide imaging for biological applications

**DOI:** 10.1186/2045-3701-4-64

**Published:** 2014-10-27

**Authors:** Hengchang Guo, Hossein Aleyasin, Bryan C Dickinson, Renée E Haskew-Layton, Rajiv R Ratan

**Affiliations:** Fischell Department of Bioengineering, University of Maryland, College Park, MD 20742 USA; Burke Medical Research Institute, Weill Medical College of Cornell University, White Plains, NY 10605 USA; Fishberg Department of Neuroscience, Friedman Brain Institute, Icahn School of Medicine at Mount Sinai, New York, NY 10029 USA; Department of Chemistry, The University of Chicago, Chicago, IL 60637 USA; School of Health and Natural Sciences, Mercy College, Dobbs Ferry, NY 10522 USA

**Keywords:** Hydrogen peroxide (H_2_O_2_), Reactive oxygen species (ROS), Molecular imaging, Fluorescent probe, Nanoparticles, Two-photon microscopy, Ratiometric imaging, Fluorescence lifetime imaging microscopy (FLIM), Chemiluminescence

## Abstract

Mounting evidence supports the role of hydrogen peroxide (H_2_O_2_) in physiological signaling as well as pathological conditions. However, the subtleties of peroxide-mediated signaling are not well understood, in part because the generation, degradation, and diffusion of H_2_O_2_ are highly volatile within different cellular compartments. Therefore, the direct measurement of H_2_O_2_ in living specimens is critically important. Fluorescent probes that can detect small changes in H_2_O_2_ levels within relevant cellular compartments are important tools to study the spatial dynamics of H_2_O_2_. To achieve temporal resolution, the probes must also be photostable enough to allow multiple readings over time without loss of signal. Traditional fluorescent redox sensitive probes that have been commonly used for the detection of H_2_O_2_ tend to react with a wide variety of reactive oxygen species (ROS) and often suffer from photostablilty issues. Recently, new classes of H_2_O_2_ probes have been designed to detect H_2_O_2_ with high selectivity. Advances in H_2_O_2_ measurement have enabled biomedical scientists to study H_2_O_2_ biology at a level of precision previously unachievable. In addition, new imaging techniques such as two-photon microscopy (TPM) have been employed for H_2_O_2_ detection, which permit real-time measurements of H_2_O_2_*in vivo*. This review focuses on recent advances in H_2_O_2_ probe development and optical imaging technologies that have been developed for biomedical applications.

**Electronic supplementary material:**

The online version of this article (doi:10.1186/2045-3701-4-64) contains supplementary material, which is available to authorized users.

## Introduction

The role of H_2_O_2_ as a second messenger, in regulating fundamental biological processes, has been identified not long ago and is increasingly supported by new data [1–7]. H_2_O_2_ is involved in therapeutic processes such as wound healing, anti-bacterial defense, stem cell proliferation, and an adaptive response in astrocytes that leads to neuronal protection [1, 5–10]. However, over-production of H_2_O_2_ exerts toxic effects on the cell and its surrounding environment. The aberrant production of H_2_O_2_ within cellular compartments is connected to serious pathological conditions such as cancer [11], ageing [12–14], diabetes [15], and neurodegenerative diseases [16–18].

A substantial challenge in elucidating the diverse roles of H_2_O_2_ in complex biological environments is measuring the spatial and temporal dynamics of this reactive oxygen metabolite. Much of the data implicating H_2_O_2_ both pathological and physiological roles in cultured cells and *in vivo* has been acquired using treatment with exogenous H_2_O_2_[19, 20], over expression of hydroperoxide inducing enzymes or H_2_O_2_ lysing/scavenging agents [1, 21], or tampering with ROS production cellular machinery such as NADPH oxidase (Nox) expressing cells [22]. Although these studies were critical in establishing crucial biological roles of H_2_O_2_, a key to fully understanding the mechanistic bases of redox biology is measuring the amount of H_2_O_2_ generated in specific intracellular compartments.

Traditional approaches to measuring H_2_O_2_ in living systems suffer from several issues: 1) The probes are often nonspecific and react with other reactive oxygen species such as hydroxyl radicals and superoxide, as well as reactive nitrogen species. 2) H_2_O_2_ is generally produced at a low concentration and can have a short half-life due to the activity of enzymes that neutralize it. 3) H_2_O_2_ is often produced in specific cellular compartments and rapidly diffuses across the membranes. For example, dichlorofluorescein (DCF), which has served as the workhorse for the redox biology community, detects multiple types of reactive small molecules, such as superoxide (O_2_^•^-), hydroperoxy radical (HO_2_^•^), singlet oxygen (^1^O_2_), peroxy radical (RO_2_^•^). DCF is not an ideal tool to determine the localization of H_2_O_2_ production. In addition to this lack of specificity of DCF for H_2_O_2_ detection, it can directly result in the creation of further ROS and thiol oxidation when exposed to UV radiation, and interacts with cytochrome c, rather than ROS as a consequence of apoptosis [23–25]. To overcome the aforementioned disadvantages for detecting ROS, recent efforts have aimed at 1) increasing the selectivity for H_2_O_2_ detection over related ROS, particularly superoxide, nitric oxide, and hydroxyl radical and 2) improving photostability and determining the localization of H_2_O_2_ production. Recently, specific and highly sensitive fluorescent H_2_O_2_ probes have been developed to circumvent these issues, including chemoselective fluorescent probes, fluorescent proteins, and nanoparticles [26, 27].

Confocal microscopy coupled with chemoselective fluorescent reporters permits the imaging of localized intracellular H_2_O_2_ levels. However, due to scattering and tissue penetration, imaging H_2_O_2_ levels in whole organisms often requires deep tissue imaging techniques. For example, two-photon microscopy (TPM) [25, 28] and photoluminescence can permit thick tissue imaging and *in vivo* studies [29, 30].

In this review, we describe and compare various methodologies for detection and imaging of H_2_O_2_ production in cells and whole organisms.

## Fluorescent probes for H_2_O_2_ imaging

### Small-molecule fluorescent probes

Small-molecule fluorescence probes for H_2_O_2_ are generally based on the oxidation–reduction processes between the H_2_O_2_ and reduced probe, which fluoresces upon oxidation. Several novel fluorescent probes capable of detecting H_2_O_2_ with high selectivity have been reported, and some of them have been used to monitor intracellular H_2_O_2_. A comprehensive list of fluorescent H_2_O_2_ probes is listed in Table 1.

**Table 1:** **Small molecule probes for H**_**2**_ **O**_**2**_ **imaging**

Fluorescent probes		Excitation/Emission (nm)	Additional features			References
Abbreviation	Full name		Detection mechanism	Intra-cellular	***In vivo***	
PX-1	Peroxyxanthone-1	350/440	Boronate-deprotection mechanism	Yes	N/A	[31]
FP-H2O2-NO	N/A	400/460	Response to H_2_O_2_, NO, or both	Yes	N/A	[32]
DMACA	N/A	400/484, 566	Ratiometric imaging	Yes	N/A	[33]
DPPEA-HC	7-hydroxy-2-oxo-N-(2-(diphenylphosphino)ethyl)-2H-chromene-3-carboxamide	403/449	PET control	N/A	N/A	[34]
PL-1	Peroxy Lucifer 1	410/475, 540	Boronate-deprotection mechanism; Ratiometric imaging	Yes	N/A	[35]
RPF-1	Ratio Peroxyfluor 1	420/464, 517	Boronate-deprotection mechanism; Ratiometric imaging	N/A	N/A	[36]
PF-1	Peroxyfluor-1	450/>460	Boronate-deprotection mechanism	Yes	N/A	[31, 37]
PF-2	Peroxyfluor-2	475/511	Boronate-deprotection mechanism	Yes	Yes	[25, 38, 39]
PF6-AM	Peroxyfluor-6 acetoxymethyl ester	482/517	Boronate-deprotection mechanism; High cellular permeability	Yes	N/A	[2, 25, 28]
NBzF	N/A	490/525	PET control	Yes	N/A	[40]
PF-3	Peroxyfluor-3	492/515	Boronate-deprotection mechanism	Yes	N/A	[38]
RF-1	Redoxfluor 1	495/503	Boronate-deprotection mechanism	Yes	N/A	[41]
MitoPY1	Mitochondrial Peroxy Yellow 1	510/528	Boronate-deprotection mechanism; Mitochondria-targeted	Yes	N/A	[25, 42, 43]
PY1-ME	Peroxy Yellow 1 Methyl-Ester	515/540	Boronate-deprotection mechanism	Yes	N/A	[44]
(NucPE) 1	Nuclear Peroxy Emerald 1	514/540	Boronate-deprotection mechanism; Nuclear localized	Yes	Yes	[45]
PY1	Peroxy Yellow 1	519/548	Boronate-deprotection mechanism	Yes	N/A	[25, 38]
PO1	Peroxy Orange 1	540/565	Boronate-deprotection mechanism	Yes	N/A	[25, 38]
Amplex Red	Amplex Red	571/581	Conjunction with horseradish peroxidase	N/A	Yes	[1, 46]
PR-1	Peroxy Red 1	~575/585	Boronate-deprotection mechanism	Yes	N/A	[31]
PCL-1	Peroxy Caged Luciferin 1	NA/612	Boronate-deprotection mechanism; Bioluminescence imaging	N/A	Yes	[30, 47]
PCL-2	Peroxy Caged Luciferin 2		Boronate-deprotection mechanism; Bioluminescence imaging	N/A	Yes	[48]
QCy7	Quinone Cy-7	595/635	Cyanine-based probe	N/A	Yes	[49]
PN1	Peroxy Naphthalene 1	TPM750nm	Boronate-deprotection mechanism	N/A	Yes	[50]
SHP-Mito	N/A	TPM750nm	Boronate-deprotection mechanism Mitochondria-targeted	Yes	Yes	[51]

**Figure 1:**
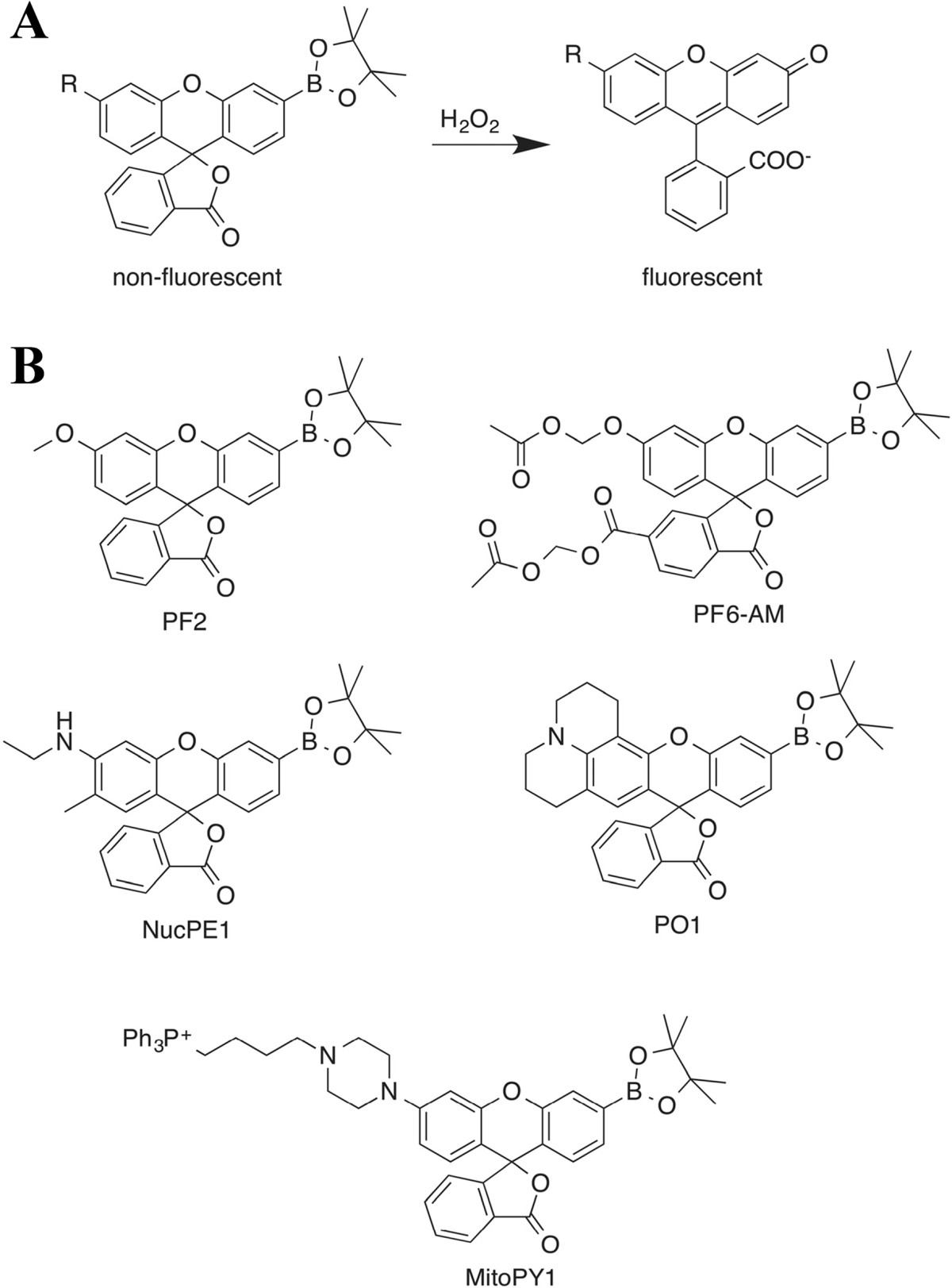
**Fluorescent turn-on mechanism and chemical structures of several examples of boronate-based H**_**2**_**O**_**2**_ **fluorescent probes. (A)** Lactone-opening mechanism of fluorescence-enhancement for mono-boronate xanthene-based H_2_O_2_ probes. **(B)** Several examples of lactone-opening-based monoboronate H_2_O_2_ fluorescent probes.

One approach for the development of H_2_O_2_-selective probes utilizes a boronate-deprotection mechanism [2–4, 31, 38, 42, 45]. Figure 1 that displays the mechanism of hydrogen peroxide-mediated fluorescent enhancement and several example structures of fluorescent probes based on this mechanism. This detection strategy relies on the selective H_2_O_2_-mediated transformation of arylboronates to phenols. Arylboronates are appended to profluorescent molecules, such that reaction with H_2_O_2_ generates a fluorescent product. The monoboronate-based family of probes (PF-1, PF-2, PF-3, PO1, and PY1) can detect physiological changes in endogenous H_2_O_2_ levels. Since a wide color palette of such probes has been developed, various combinations can be selected for multicolor imaging experiments. The addition of acetoxymethyl ester-protected pro-anionic groups gives rise to the dye peroxyfluor-6 acetoxymethyl ester (PF6-AM), which increases cellular retention and further increases sensitivity to H_2_O_2_[2, 25]. Although boronate-deprotection-based probes have improved H_2_O_2_ localization studies, quantitative analysis of H_2_O_2_ generation using fluorescent probes is still challenging. Specifically, the signal from the single-wavelength emitting probes can be affected by the concentration of the probe. To address this, a monoboronate-based probe was synthesized, Ratio Peroxyfluor 1 (RPF)-1 that provides a ratiometric change of two fluorescent signals upon reaction with H_2_O_2_, which can potentially permit normalization to probe concentration [36]. Because peroxynitrite has also been shown to react with boronates to create a fluorescent product [52], it is critical to perform proper controls when using a boronate-based fluorescent probe, such as expression of catalase or using a peroxynitite-specific probe.

Combining the boronate–based probe strategy with organelle-targetable functional groups has provided probes that can measure H_2_O_2_ levels with spatial resolution. In particular, several mitochondria-targeted probes have been generated, including Mitochondrial Peroxy Yellow 1 (MitoPY1) and SHP-Mito [25, 42, 43, 51], which utilize a positively charged phosphonium moiety for mitochondrial targeting. MitoPY1 and SHP-Mito can both be utilized as two-photon imaging probes [25, 51]. Nuclear Peroxy Emerald (NucPE) 1 is nuclear-localized fluorescent probe that utilizes a boronate protecting group to measure nuclear H_2_O_2_ levels, which has been shown to function *in vivo*[45].

All of the boronate-based probes react irreversibly with H_2_O_2,_ meaning the fluorescent signal is based on the accumulated H_2_O_2_ generated. By contrast, Redoxfluor (RF)-1 uses a reversible disulfide-based redox sensing mechanism, allowing multiple reversible redox reactions in the cellular environment to be detected over time [41]. However, one disadvantage is that RF-1 is not selective for one particular ROS.

One limitation of ROS fluorescent probes is their application in *in vivo* studies. The visible light excitation affects probe photobleaching, tissue and organ penetration, and subsequent imaging detection. To tackle these issues, Peroxy Caged Luciferin (PCL)-1 and near-infrared (NIR) probe quinone Cy-7 (QCy7) were developed [30, 47, 49]. PCL-1 and PCL-2 are prosubstrates for luciferase and have been utilized for noninvasive *in vivo* H_2_O_2_ detection throughout whole mice [30, 47, 48]. NIR imaging of the Cy-7 is another attractive tool in animal studies due to the deep penetration of the NIR photons and low background fluorescence of the tissue. QCy7 enabled monitoring H_2_O_2_ signaling upon injection of lipopolysaccharides (LPS) into mice [49].

### Nanoprobes

Compared with small-molecule fluorescent probes, nanoparticles offer several advantages [53, 54]: (1) Nanoparticles often have stronger fluorescent emission due to large number of molecular probes loaded in each single nanoprobe. (2) The high surface-area-over-volume ratio provides a higher probability for analyte detection. (3) Encapsulating small molecule fluorescent probes into nanoparticles can improve their stability. (4) The nanoparticle often serves as a protecting device for the sensory contents, protection from external interference present in biological environments (e.g., undesirable enzymatic reactions and nonspecific uptake by proteins). (5) Nanoprobes can possess multifunctionality, and target-specificity by conjugating ligand moieties onto the nanoparticle surface.

A number of nanoprobes were listed in Table 2. Peroxalate-based nanoparticles were recently developed which chemically excites the encapsulated dye, leading to light emission from the nanoparticles and the imaging of H_2_O_2_[29, 55–57]. Encapsulating fluorescent probes into nanoparticles can improve their stability. This nanoprobe was recently demonstrated by Lee et al. for *in vivo* imaging of global H_2_O_2_ in mouse model [29]. Semiconducting polymer-based nanoprobe CF-SPN has another advantage that combines chemiluminescence imaging with ratiometric imaging for liver-targeted detection of ONOO- and H_2_O_2_ simultaneously in the liver of living mice and in real time [58]. This nanoprobe demonstrated multifunctionality chemiluminescence imaging and ratiometric imaging for ROS sensing. For high sensitivity single-molecule detection, single-walled carbon nanotube (SWNT) embedded nanosensor exhibited high selectivity and sensitivity to single molecules of H_2_O_2_[59–61], which raises the potential for a hitherto unseen level of specificity in redox signaling analysis.

**Table 2:** **Nanoparticles for H**_**2**_ **O**_**2**_ **detection**

Nanoparticles	Detection features	References
Peroxalate nanoparticles	Chemiluminescence imaging	[29, 55–57]
FPOC NPs	Chemiluminescence imaging;	[62]
CF-SPN	Chemiluminescence imaging; Ratiometric imaging	[58]
TiO_2_ nanorods	Confocal microscopy; HRP-catalyzed oxidation	[63]
NanoPEBBLE	Confocal microscopy; Hydrophobic Ormosil Nanoparticles	[64]
HRP-loaded PEG hydrogel spheres	Fluorescence imaging; Oxidized fluorophores such as Amplex Red becoming fluorescence.	[65]
SWNT	Photoluminescence detection; Single molecule sensitivity	[59–61]
Fe_3_O_4_	Magnetic Nanoparticles	[66, 67]
Gold (Au) Nanodot	Luminescence quenching	[68]
Cyclometalated Iridium(III)	Phosphorescent probe	[69]

### Genetic fluorescent proteins

In the past, researchers have largely relied on the use of fluorescent dyes for ROS sensing due to their good sensitivity, high signal-to-noise ratio, cell permeability, and ease of measurement. The emerging genetic fluorescent proteins offer another option for high resolution selective H_2_O_2_ imaging. Allowing a dynamic measurement for the reversible detection of H_2_O_2_, Belousov, et al. developed a group of genetically encoded fluorescent proteins HyPer and their mutants to enable transient live-cell imaging [6, 70–75]. It is a ratiometric fluorescent indicator of H_2_O_2_ in which cpYFP is inserted into the regulatory domain of an Escherichia coli peroxide sensor OxyR [71]. HyPer is able to detect nanomolar concentrations of H_2_O_2_*in vitro*, to micromolar levels of H_2_O_2_ exogenously added to cells, or changes of intracellular H_2_O_2_ upon growth factor stimulation [71]. Due to genetic labeling, the HyPer family of genetically encoded fluorescent proteins have been successfully targeted to several cellular compartments such as the nucleus, cytosol, peroxisomes, mitochondria and the endoplasmic reticulum [70]. Thereby they allow for the intracellular spatial monitoring of H_2_O_2_ production, which can further improve H_2_O_2_ imaging with high signal-to-background noise from tissue auto-fluorescence in biological systems. HyPer probes are pH sensitive. To address this issue pH-specific probes are used as controls. HyPer-C199S would be an ideal control as a pH-sensitive and H_2_O_2_-insensitive version [76].

To improve the dynamic range of half-oxidation and half-reduction responses, HyPer-2 and HyPer-3 were developed which shows an expanded dynamic range. HyPer-3 showed faster oxidation-reduction kinetics and a higher fluorescence ratio (F500/F420) than what was reported for HyPer, demonstrating its advantage for H_2_O_2_ detection [77]. Both HyPer and HyPer-3 are applicable for fluorescence lifetime imaging microscopy (FLIM).

Redox-sensitive GFP (roGFP) [72, 73] coupled with yeast H_2_O_2_-sensing signaling peroxidase Orp1 [78] is a genetically encoded H_2_O_2_ sensor that has been used to detect and quantify physiological levels of H_2_O_2_*in vivo*. In roGFP2-Orp1, Orp1 relays a disulfide bridge to redox-sensitive GFP (roGFP). The redox equilibrium of the engineered cysteines is associated with measurable ratiometric fluorescent changes. Contrary to HyPer probes, this redox-dependent fluorescence is insensitive to pH changes in the physiological range. RoGFP2-Orp1 has been successfully used to measure physiologically relevant changes in H_2_O_2_ levels in Drosophila tissues and in living larvae by real-time imaging [79–81].

## Optical imaging of H_2_O_2_ in biological system

### Confocal microscopy

Confocal microscopy uses a scanning laser beam that is focused on the sample for imaging, with a pinhole placed in front of the detector. Confocal microscopy increases optical resolution and contrast by using a pinhole that prevents the out-of-focus photons from reaching the detector. It is the most popular imaging technique for H_2_O_2_ detection *ex vivo*. Most fluorescent probes were developed for confocal microscopy. However, confocal microscopy has limitations, such as optical scattering, photodamage, photobleaching, and limited imaging depth for use in real time *in vivo* studies. Furthermore, prolonged visible light exposure can result in artifactual ROS generation and signal amplification [82].

The recent advance of *in vivo* confocal microscopy is based on microendoscopy. By utilizing a miniprobe for confocal microendoscopy, PY1 was demonstrated for *in vivo* H_2_O_2_ imaging in colorectal cancer [83].

### Two-photon microscopy

TPM provides sub-micron resolution imaging with lower phototoxicity and deeper tissue penetration than confocal microscopy [28]. In the two-photon process, a molecule simultaneously absorbs two photons whose individual energy is only half of the energy needed to excite that molecule, and then releases the energy to an emission photon. The main differences between confocal microscopy and TPM are the excitation light source and the fluorescence detection unit. TPM, including all commercial versions, is typically implemented in a laser scanning microscope equipped with a NIR ultrafast pulse laser.

**Figure 2:**
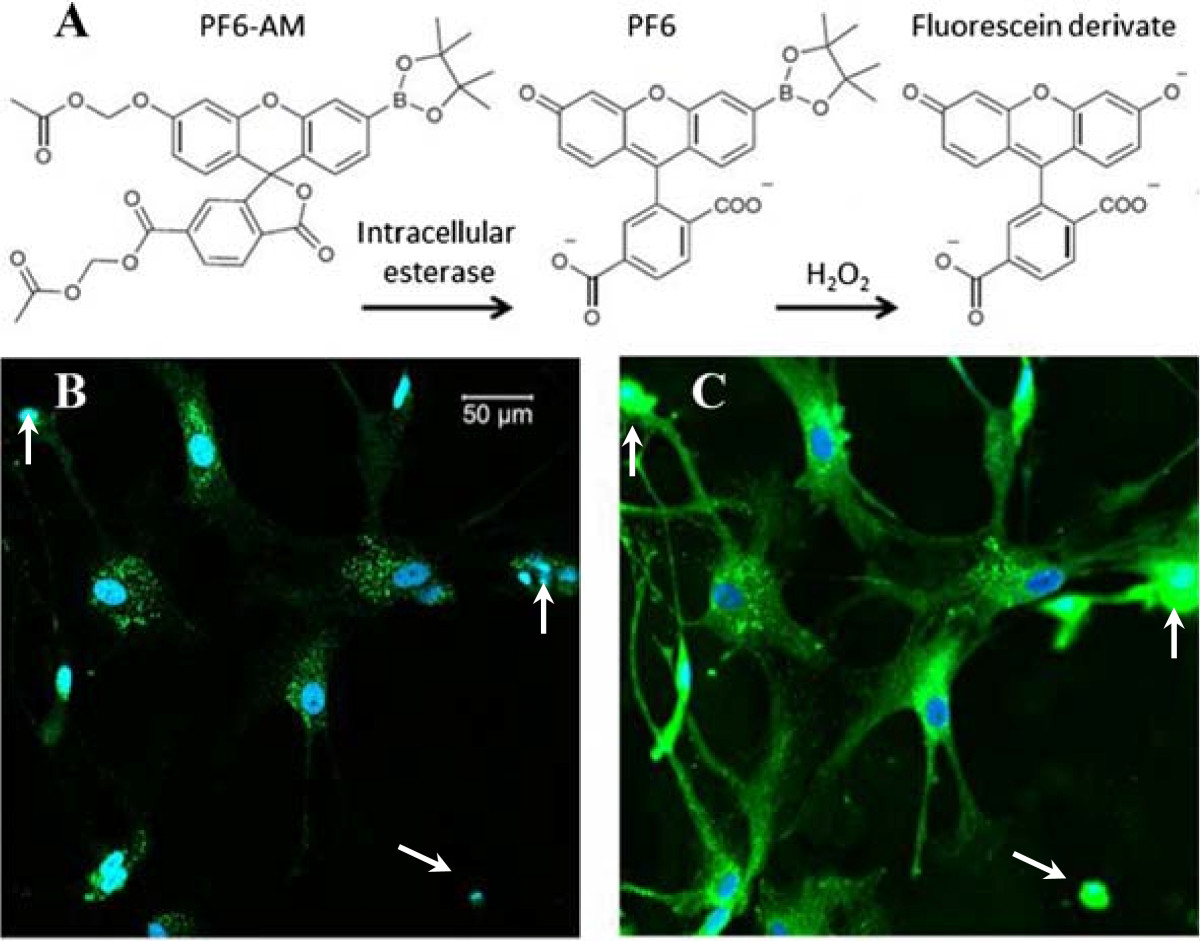
**Fluorescence imaging of intracellular H**_**2**_**O**_**2**_ **production using fluorescence probe PF6-AM (green). (A)** Mechanism of Chemoselective H_2_O_2_ PF6-AM. **(B)** TPF imaging of H_2_O_2_ in astrocytes, fluorescence excited with a 770 nm Ti:sapphire laser. **(C)** Confocal microscopy of H_2_O_2_ in same astrocytes imaged in panel **B**, fluorescence excited with a 488 nm laser. The nuclei were stained with Hoechst 33342 (blue).

TPM was demonstrated for imaging intracellular H_2_O_2_ production in live cells and tissues [25, 35, 50, 51]. Figure 2 shows TPM imaging of intracellular H_2_O_2_ in rat primary astrocytes using the chemoslective fluorescence probe PF6-AM. Figure 2A shows the H_2_O_2_ imaging mechanism of trappable probe PF6-AM [2]. Figure 2B shows TPM imaging of intracellular H_2_O_2_. As a comparison, Figure 2C shows confocal microscopy of the same cells using a 488 nm argon laser with the same fluorescence detection. Three arrows indicate strong light scattering in the same cells in Figure 2C. The TPM imaging here demonstrated the advantages of low scattering and low background noise.

For the deep tissue *in vivo* TPM, it has motivated new trends of technology development including long wavelength lasers [84–88], fast scanner [89, 90], and microendoscopes [91–93]. These techniques may further extend *in vivo* TPM for deep tissue H_2_O_2_ imaging in real time.

### Ratiometric imaging

Ratiometric imaging is the division of one fluorescence channel by another one to derive the ratiometric channel. Ratiometric imaging has been widely used to detect intracellular ion concentrations, protein distributions, voltage or pH changes [94]. Compared to traditional fluorescence intensity imaging, ratiometric imaging relies on measuring a shift in emission instead of merely a change in intensity. It is extremely attractive for quantitative analysis because it corrects for unequal fluorophore labeling and photo-bleaching. Dual wavelength excitation/detection is the key for measuring emission shifts and intensity changes of fluorophores. The recent development of ratiometric H_2_O_2_ probes have been used for ratiometric imaging based on wide-field microscopy, confocal microscopy and TPM [35, 50, 51, 71, 77, 94–96].

**Figure 3:**
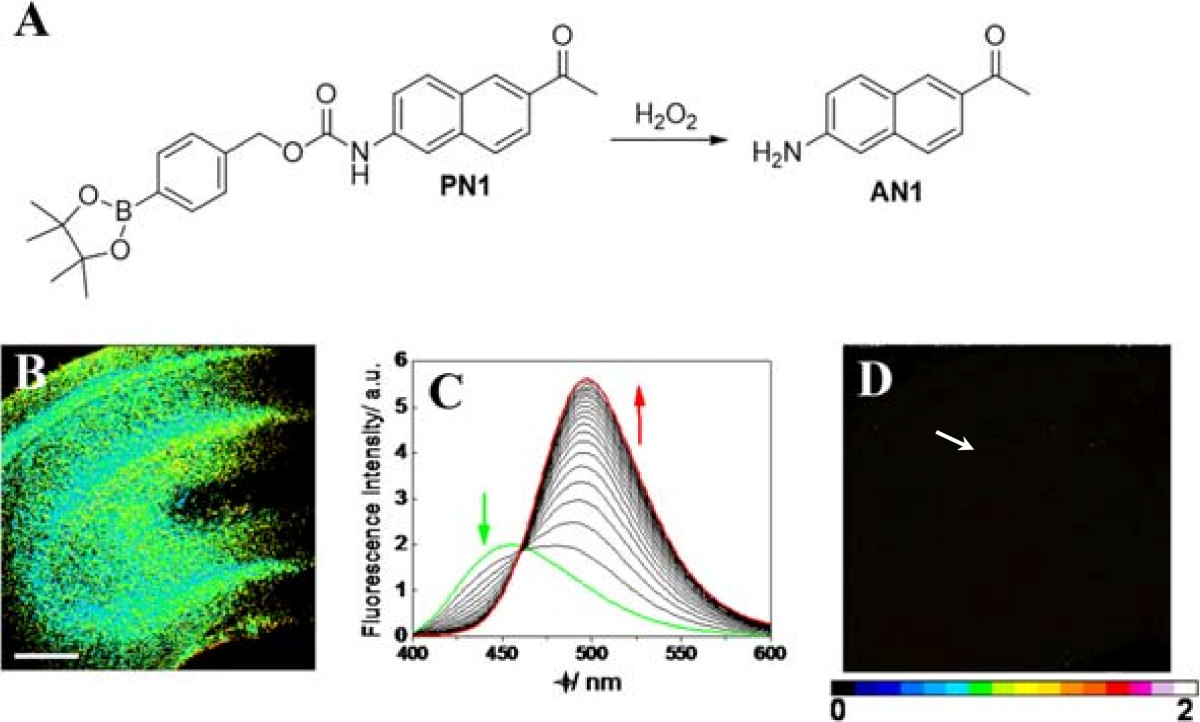
**Ratiometric imaging of fresh rat hippocampal slice treated with H**_**2**_**O**_**2**_**. (A)** The reaction between PN1 and H_2_O_2_ produced AN1 as the only major fluorescent product. **(B)** A hippocampal slice labeled with PN1. **(C)** Fluorescence spectra responses of 3 μM PN1 to 1 mM H_2_O_2_. Spectra were acquired at 0, 10, 20, 30, 40, 50, 60, and 120 min after H_2_O_2_ was added. **(D)** A hippocampal slice labeled with PN1 after pretreated with H2O2. Scale bars: 30 μm. The figures were adapted from ref. [50] with permission.

Figure 3 shows TPM ratiometric image of a fresh rat hippocampal slice treated with H_2_O_2_ production. This imaging technique provides a solution for deep tissues H_2_O_2_ quantitative analysis.

### FLIM

**Figure 4:**
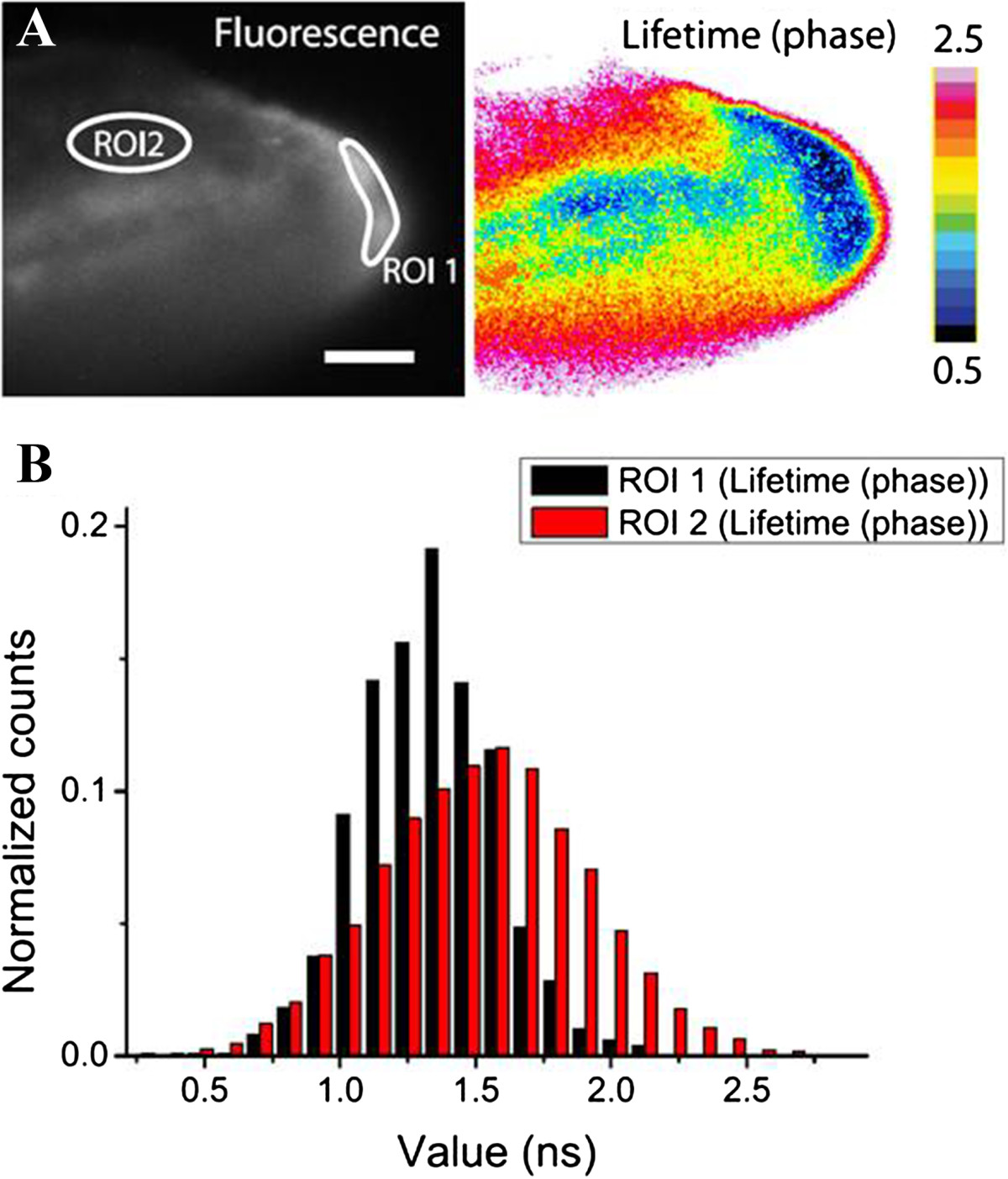
**FLIM of HyPer-3 response to H**_**2**_**O**_**2**_ **production induced by inflammation in zebrafish larvae. (A)** Left and right panels represent fluorescence intensity and FLIM images, respectively. ROI1 highlights the wound margin; ROI2 represents an area distant from the wound. **(B)** Fluorescence lifetime distribution plot for ROI1 and ROI2 in panel **A**. The figures were adapted from ref. [77] with permission.

FLIM is an optical imaging technique based on the differences in the exponential decay rate of the fluorescence from a fluorescent sample [97, 98]. Because fluorescence lifetime τ is independent of indicator concentration, FLIM measurement is essentially insensitive to indicator expression level, non-uniform distribution, and partial photobleaching. FLIM generates absolute quantitative readouts while requiring only a single-wavelength excitation, provided that the indicator is calibrated *in situ* (e.g., in permeablized cells) or *in vitro* under conditions closely resembling intracellular environments. Figure 4 shows inflammation driven H_2_O_2_ production in zebra fish larvae using representative FLIM of HyPer-3 [77]. The pattern of the fluorescence lifetime changes indicated the gradient of H_2_O_2_ with higher concentrations of the oxidant at the wounding site. This imaging technique provides another solution for H_2_O_2_ quantitative analysis.

### Chemi-/bioluminescence imaging

*In vivo* chemi-/bioluminescence imaging is a popular method to monitor enzymatic light emission by a living organism [99–101]. The detection signal is generally from red to near-infrared (NIR) light that offers deep depth imaging of H_2_O_2_ in organs. By using cooled charge-coupled device (CCD) cameras, this optical imaging modality gets high sensitive. The field of view is up to scores of centimeter covering a whole small animal, but the resolution is at the millimeter level. The recent advances of bioluminescence and chemiluminescence probes enable whole animal studies of H_2_O_2_ production [29, 30, 47–49, 55–57, 62, 102].

**Figure 5:**
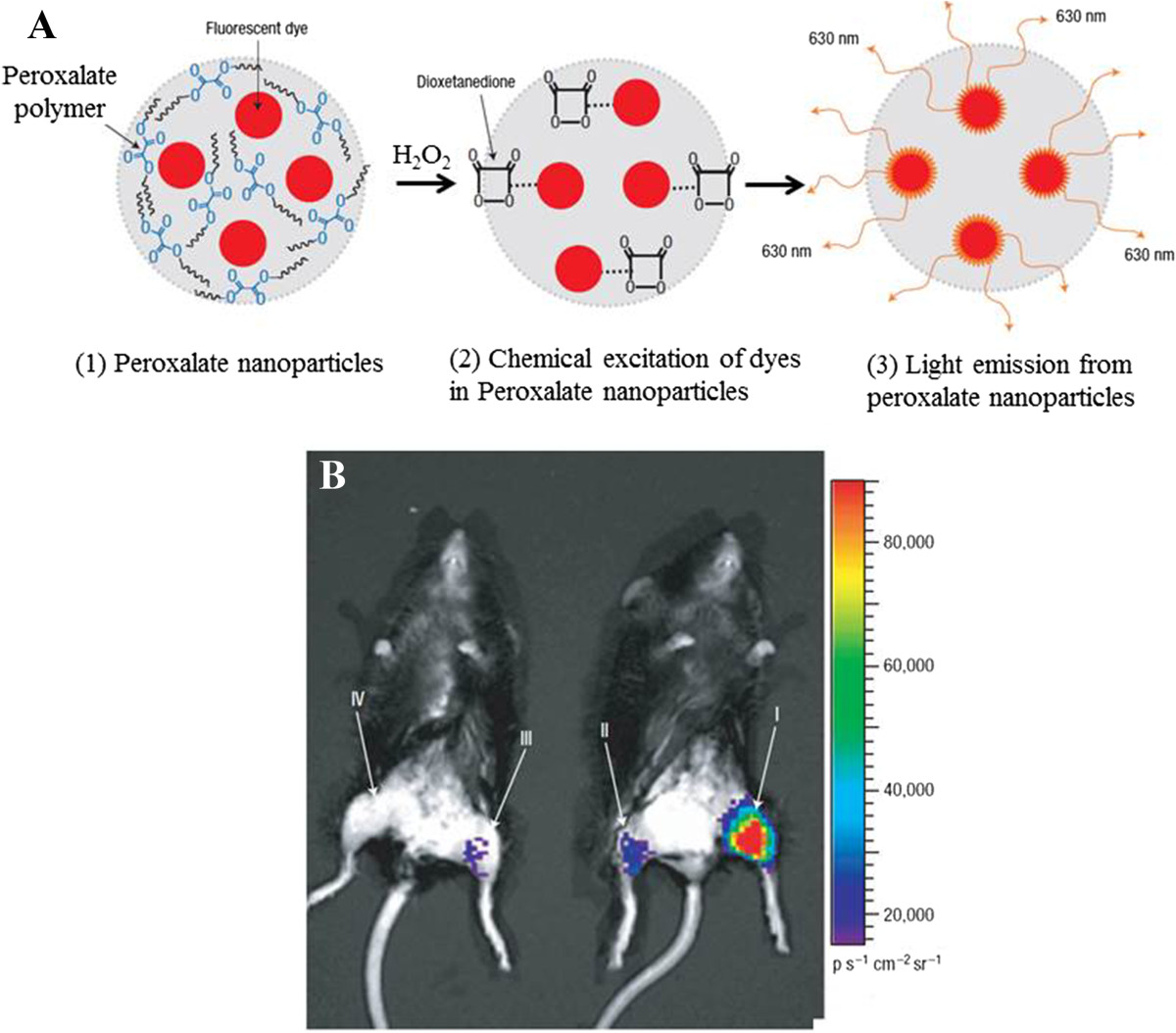
***In vivo*** **imaging of H**_**2**_**O**_**2**_ **using peroxalate nanoparticles. (A)** Peroxalate nanoparticle. **(B)** Chemiluminescence imaging. The figures were adapted from ref. [29] with permission.

Figure 5 shows representative chemiluminescence images of global H_2_O_2_ production in a mouse model using Peroxalate nanoparticles [29]. H_2_O_2_ reacts with the peroxalate ester of (1) Peroxalate nanoparticles to produce a high-energy dioxetanedione intermediate within the nanoparticles (2), which then chemically excites the encapsulated dye, leading to light emission from the nanoparticles (3). Peroxalate nanoparticles were mixed with various concentrations of H_2_O_2_ and injected, intramuscularly, into the legs in Figure 5B. The concentration is (I) 10 μM H_2_O_2_, (II) 1 μM H_2_O_2_, (III) peroxalate nanoparticles only, and (IV) negative control. The chemi-/bioluminescence imaging is the only technique for whole animal global H_2_O_2_ monitor in real time.

## Conclusion

It is imperative to develop fluorescent probes that are able to monitor spatio-temporal intracellular H_2_O_2_ production in real time for live cells and *in vivo* studies. Among these fluorescent probes, chemoselective probes offer an attractive approach to H_2_O_2_ detection due to their general compatibility with an array of biological systems without external activating enzymes and genetic manipulation. Nanoprobes were recently developed for *in vivo* imaging of H_2_O_2_. A benefit arising from the multifunctional nanotechnology, probe is that it is designed for both chemiluminescence imaging and ratiometric imaging. Furthermore, genetic fluorescent probes that target redox sensitive proteins to specific cellular locations provide high sensitive targeted imaging technology for real time H_2_O_2_ imaging.

These emerging probes enable H_2_O_2_ detection using: 1) high resolution fluorescence imaging such as confocal microscopy and TPM; 2) large field of view global imaging involving *in vivo* chemi-/bioluminescence imaging; and 3) ratiometric imaging or FLIM for the quantification of cellular H_2_O_2_ levels.

## Authors’ original submitted files for images

Below are the links to the authors’ original submitted files for images. Authors’ original file for figure 1 Authors’ original file for figure 2 Authors’ original file for figure 3 Authors’ original file for figure 4 Authors’ original file for figure 5

## Abbreviations

DCF: Dichlorofluorescein
FLIM: Fluorescence lifetime imaging microscopy
H_2_O_2_: Hydrogen peroxide
NIR: Near-infrared
PEG: Polyethylene glycol
PET: Photoinduced electron transfer
ROI: Region of interest
ROS: Reactive oxygen species
SWNT: Single-walled carbon nanotube
TPM: Two-photon microscopy.
