# Supplementary figures and images for: Recent advances in hydrogen peroxide imaging for biological applications
Source: Cell Biosci. 2014 Oct 27;4:64. doi: 10.1186/2045-3701-4-64 (PMC4232666; doi:10.1186/2045-3701-4-64)

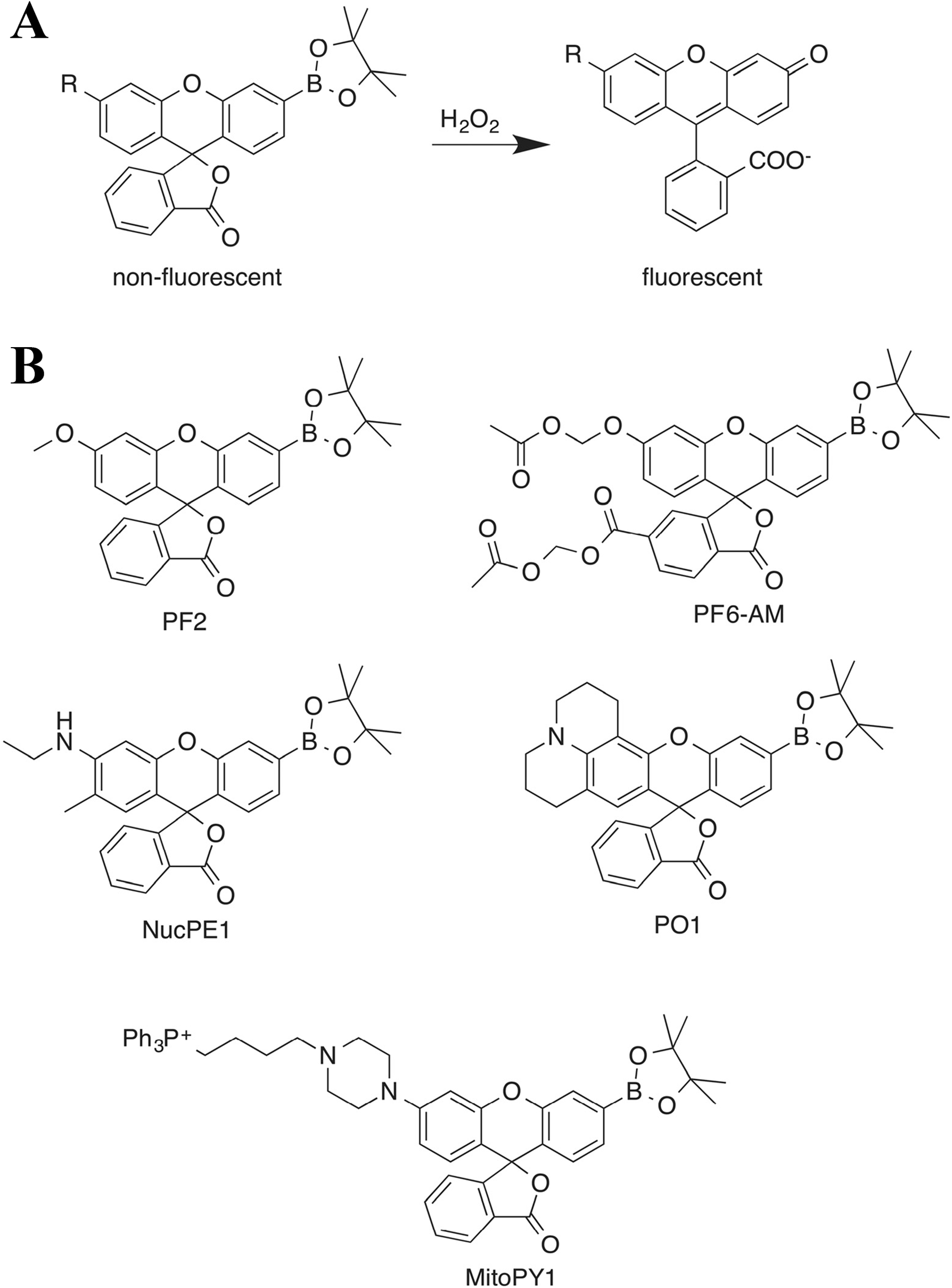

Supplement: Supplementary file 1 — Authors’ original file for figure 1 [file 13578_2014_191_MOESM1_ESM.tif]

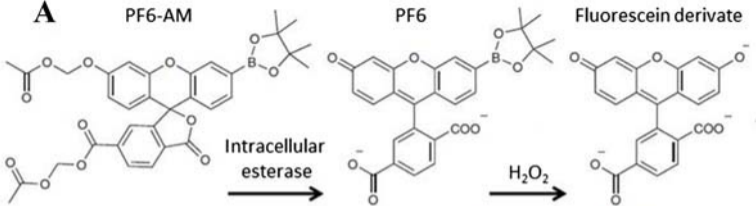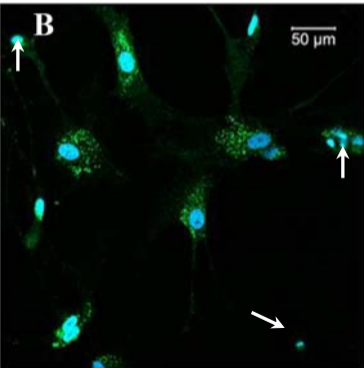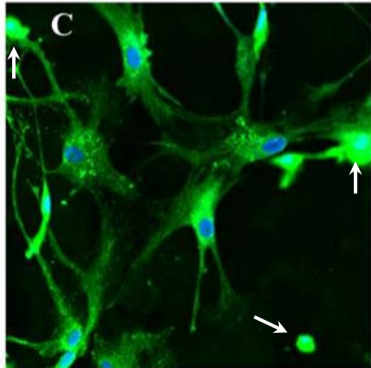

Supplement: Supplementary file 2 — Authors’ original file for figure 2 [file 13578_2014_191_MOESM2_ESM.pdf]

**A**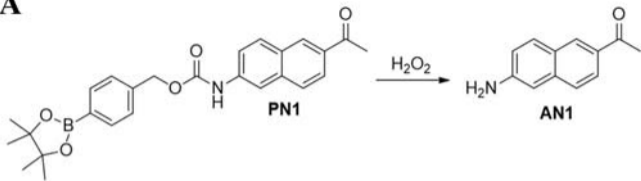**B**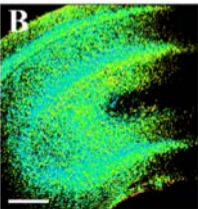**C**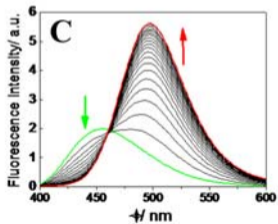**D**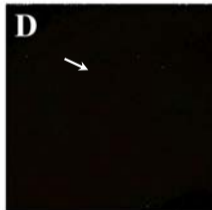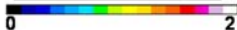

Supplement: Supplementary file 3 — Authors’ original file for figure 3 [file 13578_2014_191_MOESM3_ESM.pdf]

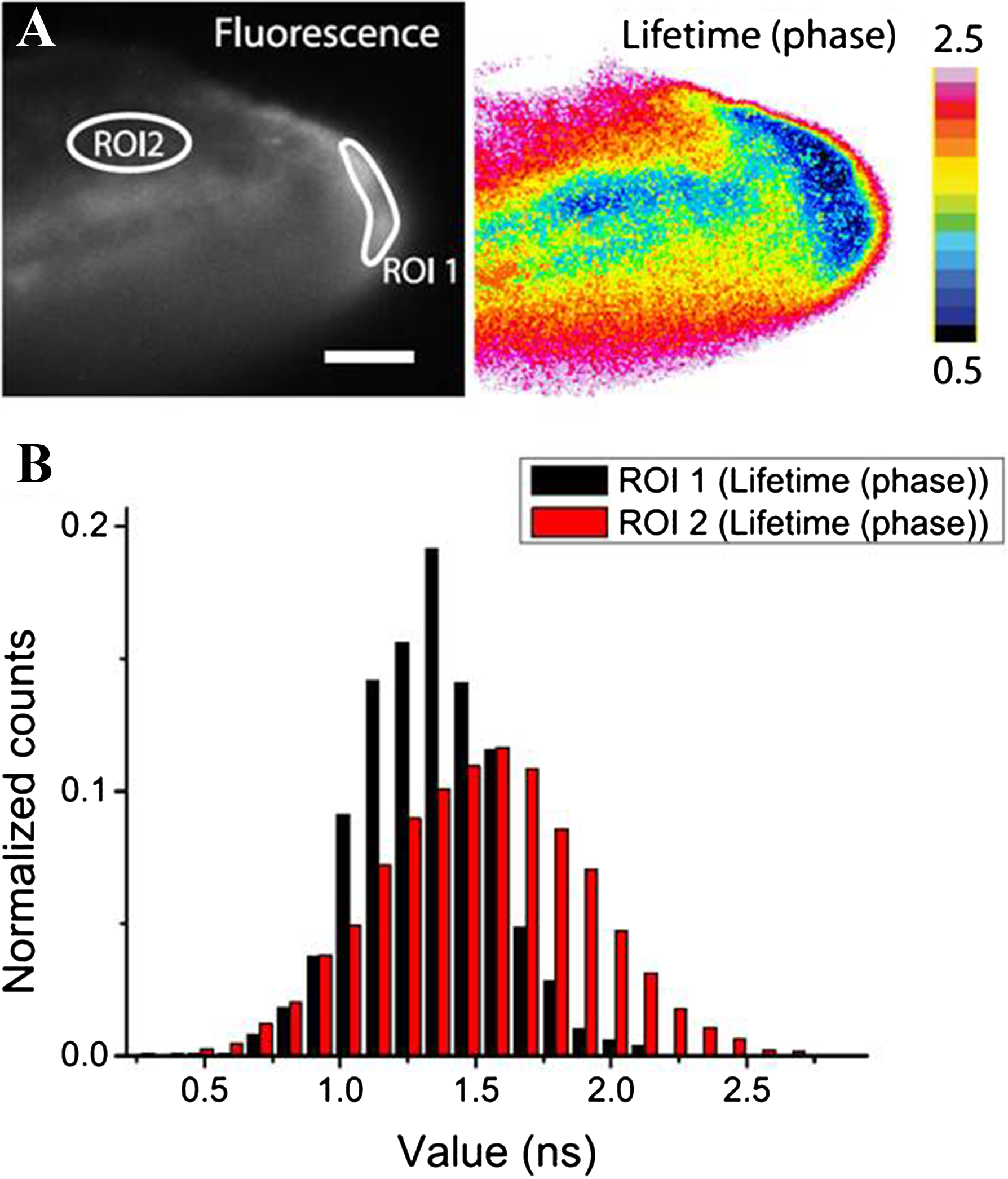

Supplement: Supplementary file 4 — Authors’ original file for figure 4 [file 13578_2014_191_MOESM4_ESM.tif]

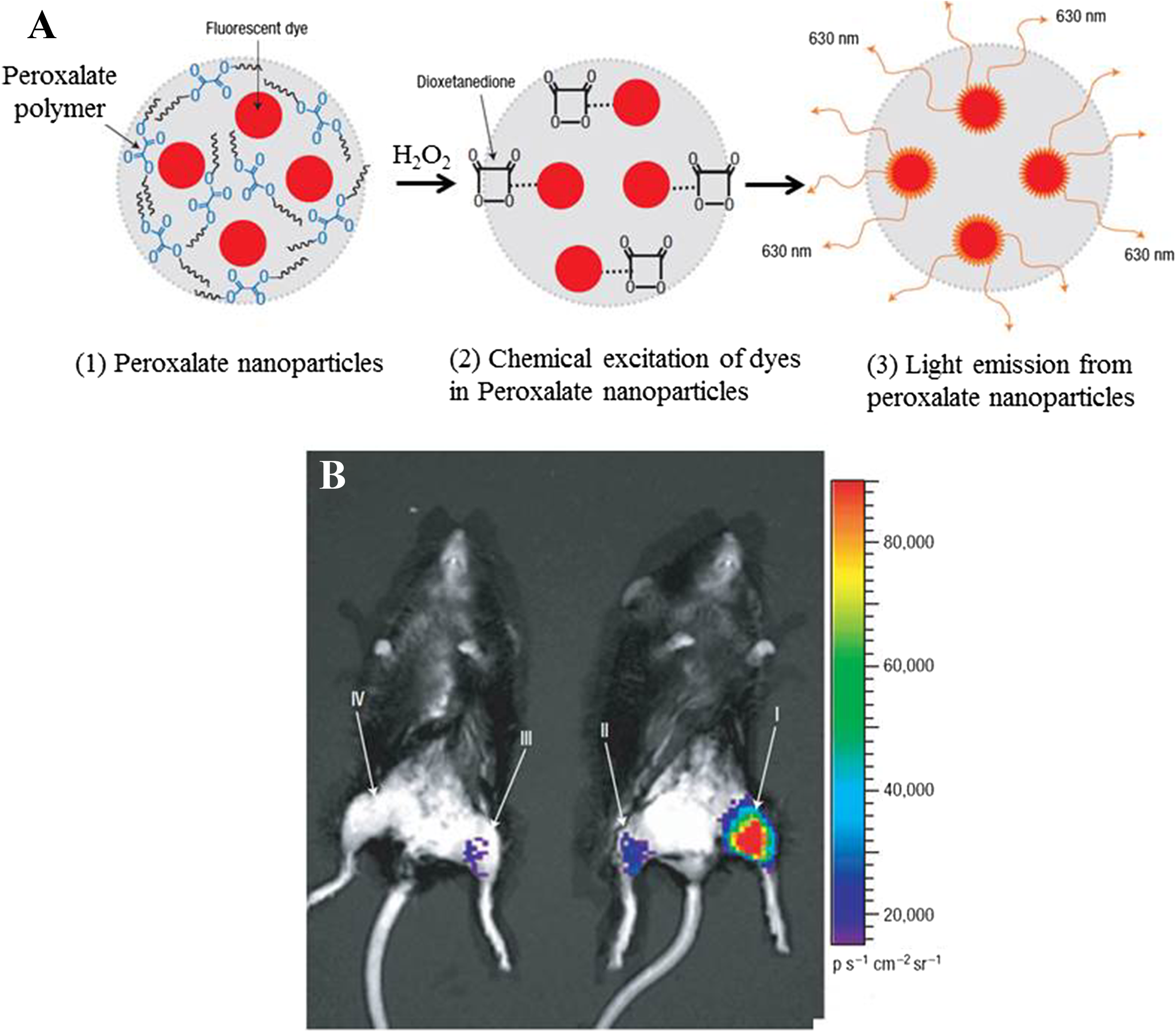

Supplement: Supplementary file 5 — Authors’ original file for figure 5 [file 13578_2014_191_MOESM5_ESM.tif]
